# Mortality trends and influencing factors of mental disorders among urban and rural residents in China between 2009 and 2021: a longitudinal study with projections to 2030

**DOI:** 10.3389/fpubh.2026.1851033

**Published:** 2026-09-03

**Authors:** Kaiyao Shen, Chenghao Wu, Wenhui Kong, Xu Wang, Yujie Zhao, Xueying Wang, Shan Hong, Hui Li

**Affiliations:** School of Public Health, Jilin University, Changchun, China

**Keywords:** aging, Grey forecasting model, influencing factors, mental disorders, mortality trends, urban-rural disparities

## Abstract

**Introduction:**

Mental disorders pose a major public health challenge in China, with marked urban–rural disparities in resources and outcomes. However, long-term mortality trends, their influencing factors, and future projections across urban and rural areas remain underexplored. This study aimed to analyze these mortality trends, identify relevant factors, and project mortality to 2030 using national data from 2009 to 2021.

**Methods:**

Data were extracted from the *China Health Statistical Yearbook*, the *China Health and Family Planning Statistical Yearbook*, the *China Health Statistics Yearbook* and the *China Statistical Yearbook* covering the years 2009–2021. Joinpoint regression was used to analyze temporal trends. A two-way fixed effects panel regression model examined associated factors, with additional sensitivity analyses including Oster’s boundary analysis and the exclusion of dementia-related deaths. GM (1,1), ARIMA, and Lasso models were compared for forecasting, and optimistic and pessimistic scenarios were constructed to reflect uncertainty in projections to 2030.

**Results:**

From 2009 to 2021, urban crude mortality rates (CMR) remained stable, whereas rural CMR increased significantly (AAPC = 1.21%, *p* = 0.02), with a sharp rise during 2019–2021 (APC = 13.23%, *p* < 0.01). Age-standardized mortality rates (ASMR) declined only in rural areas (AAPC = −3.88%, *p* < 0.01). Males had higher ASMR than females, and the oldest age groups bore the heaviest burden. The 20–25 years age group showed an increase from 2018 to 2021, although this trend did not reach statistical significance. Aging rate was the strongest factor associated with ASMR (*β* = 0.650, *p* < 0.01), and this association persisted after controlling for mental disorder prevalence, suicide-specific mortality, and lifestyle factors. An Oster boundary analysis indicated that unmeasured confounders would need to be substantially stronger than observed covariates to eliminate this association. Scenario projections suggested continued divergence between urban and rural trends, with a wide range between optimistic and pessimistic scenarios.

**Conclusion:**

This study reveals significant urban–rural disparities in mental disorder mortality, with rural areas experiencing a marked recent increase. Males, the oldest old, and young adults are particularly vulnerable. Population aging showed the strongest association with mortality, and this association persisted after controlling for additional confounders. Scenario projections indicated continued divergence between urban and rural trends. These findings underscore the need for life-course prevention strategies and differentiated resource allocation between urban and rural areas.

## 1 Introduction

Mental disorders represent a major component of the global disease burden, contributing substantially to disability-adjusted life years (DALYs) and socioeconomic costs. According to the Global Burden of Disease Study 2019, mental, and substance use disorders accounted for over 125 million DALYs worldwide, establishing them as leading causes of health loss among non-communicable diseases (1). Beyond direct health impacts, these conditions impose considerable economic costs, with approximately half of the associated social burden stemming from indirect losses such as reduced productivity and premature mortality. Without effective interventions, mental disorders are projected to cost the global economy up to US$16 trillion by 2030 (2).

In China, rapid socioeconomic transformation has increasingly brought mental health issues to the forefront. National surveys indicate that, excluding dementia, the 12-month and lifetime prevalence rates of mental disorders among Chinese adults are 9.3 and 16.6%, respectively, with depressive symptom detection rates exceeding 10% in certain subgroups (3). This high burden translates into substantial economic costs, estimated at US$88.8 billion in 2024 (4). Moreover, mental disorders frequently co-occur with physical illnesses, leading to complex disease interactions and adverse outcomes (5).

Since the implementation of the Central Government Subsidy for Local Health Services—Severe Mental Disorder Management and Treatment Program in 2004, China has made considerable efforts to strengthen its mental health service system. The enactment of the *Mental Health Law of China* in 2013 and the ‘*Healthy China 2030’* initiative in 2016 provided legal and strategic foundations for the prevention and treatment of mental disorders (6, 7). Despite these advances, challenges remain, particularly the uneven distribution of mental health resources between urban and rural areas. For instance, the density of psychiatrists in urban areas is approximately 1.5 times that in rural areas, with even greater disparities observed for specialized professionals such as psychotherapists (8, 9). Such inequities may contribute to urban–rural disparities in mortality outcomes among patients with mental disorders. Nevertheless, the trends in mortality from mental disorders across urban and rural in China, as well as the factors driving trends, have not been systematically examined.

Given this context, this study systematically analyzed the trends and associated influencing factors of mortality from mental disorders among Chinese urban and rural residents from 2009 to 2021. We identified key transition points in mortality trends and quantified the contributions of healthcare resources, socioeconomic conditions, and demographic structure. Additionally, we projected mortality trends up to 2030. The findings aim to provide scientific evidence for the prevention and control of mental disorders and for the optimal allocation of mental health resources.

## 2 Materials and methods

### 2.1 Data sources

All data used were extracted from the *China Health Statistical Yearbook*, the *China Health and Family Planning Statistical Yearbook*, the *China Health Statistics Yearbook* and the *China Statistical Yearbook* from the period 2009–2021 (10). And deaths from mental disorders were identified according to ICD-10, with codes ranging from F00 to F99.

The indicators extracted includes (1) Mortality indicators: crude mortality rate (CMR) for mental disorders by urban and rural areas, sex, and age group, covering age range from infancy (under 1 year) to the oldest-old (aged 85 years and above); cause-specific mortality composition ratio by urban and rural areas; suicide-specific mortality. (2) Healthcare resource indicators: number of psychiatrists, psychiatric beds, psychiatric outpatient and emergency visits, psychological counselors, and community health service centers, all expressed as densities per 100,000 population. Psychiatrist workload was defined as the annual number of psychiatric outpatient and emergency visits per psychiatrist. (3) Socioeconomic indicators: per capita disposable income, basic medical insurance coverage rate, and urbanization rate. (4) Demographic and epidemiological indicators: aging rate, defined as the proportion of the population aged 65 years and above; population-level prevalence of mental disorders. (5) Lifestyle indicators: adult smoking rate and per capita alcohol consumption (11, 12).

### 2.2 Statistical analysis

#### 2.2.1 Data processing

Based on the extracted indicators, age-standardized mortality rate (ASMR) was calculated using the 2010 national census population as the standard, and healthcare resource densities were calculated as counts per 100,000 population for each year and area. For age groups under 20 years, sporadic missing values were imputed using linear interpolation based on adjacent years’ observations; values at the beginning or end of the time series that could not be interpolated were set to zero, consistent with the extremely low mortality from mental disorders in these age groups. Differences in mortality rates across regions and sexes were compared using the Mann–Whitney *U* test and independent samples *t*-test, respectively. *p* < 0.05 was considered statistically significant.

#### 2.2.2 Joinpoint regression analysis

Joinpoint regression was used to analyze temporal trends in mortality indicators and identify years with significant trend changes. A log-linear model was fitted with the natural logarithm of the mortality rate as the dependent variable and calendar year as the independent variable (13). The optimal number of joinpoints was determined by permutation tests, with a maximum of three joinpoints allowed. Annual percentage changes (APC) and average annual percentage changes (AAPC) were calculated with 95% confidence intervals. An APC or AAPC greater than 0 indicates an increasing trend, and a value less than 0 indicates a decreasing trend (14).

#### 2.2.3 Panel regression analysis

A two-way fixed effects panel regression model was employed to examine factors associated with ASMR. The model included year fixed effects and a dummy variable for urban residence, estimated using ordinary least squares (OLS) with robust standard errors (15, 16). This specification controls for unobserved time-invariant differences between urban and rural areas while accounting for common temporal shocks affecting both areas. Given the binary urban–rural classification, the area fixed effect is equivalent to a single dummy variable. The Durbin-Watson test was used to assess autocorrelation and values close to 2 indicate no significant autocorrelation (17). And multicollinearity was assessed using variance inflation factors (VIF). A VIF value below 5 was considered indicative of no serious multicollinearity.

A stepwise modeling strategy was applied. Model 1 included only healthcare resource variables; Model 2 added socioeconomic factors; Model 3 additionally incorporated the aging rate; and Model 4 further included mental disorder prevalence, suicide-specific mortality, adult smoking rate, and per capita alcohol consumption. To compare the relative importance of predictors, standardized regression coefficients were calculated by standardizing all variables to a mean of 0 and a standard deviation of one prior to estimation.

In addition, sensitivity analyses were conducted to further assess the robustness of the findings. Models 1–4 were re-estimated using data from 2009 to 2019 only, and Model 4 was re-estimated for the 2013–2021 period with non-dementia ASMR as the dependent variable.

#### 2.2.4 Robustness to omitted variable bias

To quantify the potential impact of unmeasured confounders on the estimated coefficients as well, we applied Oster’s (2019) method to assess sensitivity to unobserved confounders. Comparing a restricted model and a full model, we computed for each coefficient the required selection ratio δ that would drive the estimate to zero (18):


$$\delta =\frac{\left(\beta_{r}-\beta_{f})(R_{\max}^{2}-R_{f}^{2}\right)}{\beta_{f}(R_{f}^{2}-R_{r}^{2})}$$

where $\beta_{r}$ and $\beta_{f}$ are the restricted and full-model coefficients, $R_{r}^{2}$ and $R_{f}^{2}$ the corresponding $R^{2}$ , and $R_{\max}^{2}=1.3\;R_{f}^{2}$ . A larger δ implies greater robustness to omitted variable bias (19). And we used Model 3 as the restricted model, and Model 4 as the full model. For variables already present in Model 3, we calculated δ to assess their sensitivity to omitted variable bias. For variables newly added in Model 4, the formula would yield a negative δ ; therefore, we do not report δ for these variables. Instead, we report the bias-adjusted coefficient $\beta^{*}$ assuming unobservables are as important as observables ( δ=1 ).

#### 2.2.5 Mortality projections and scenario analysis

Three forecasting models were compared GM (1,1), ARIMA with automatic order selection, and Lasso regression. Models were trained on 2009–2017 data and evaluated on 2018–2019 data to avoid potential bias from pandemic related mortality shocks. Model performance was assessed using root mean square error (RMSE) and mean absolute error (MAE). ARIMA models were fitted with maximum orders of (2,1,2), and the optimal (p,d,q) parameters were selected by minimizing the Akaike information criterion (AIC) (20). Lasso regression used year as the predictor and the regularization parameter α was selected via leave-one-out cross-validation (21).

The selection of the baseline forecasting model was guided by the ability to generate directional projections and broad alignment with the trend breaks identified by Joinpoint analysis. The model best satisfying these considerations was used for the baseline projections. All three models were fitted to the full 2009–2021 series to generate projections to 2030. The median forecast across the three models was calculated for each projection year, and optimistic and pessimistic scenarios were constructed as 90 and 110% of this three model median, respectively, reflecting a plausible range of policy and demographic trajectories.

### 2.3 Statistical software

Data preprocessing was conducted using Excel 2019. Joinpoint regression analyses were performed using Joinpoint Regression Program version 4.9.1.0. Panel regression analyses, missing value imputation, and forecasting model fitting with scenario projections were performed using Python 3.12.4.

## 3 Results

### 3.1 Descriptive analysis

#### 3.1.1 Overall mortality levels

From 2009 to 2021, the CMR for mental disorders in urban areas fluctuated between 2.00 and 3.60 per 100,000, while in rural areas it increased from 3.08 to 3.54 per 100,000. The ASMR was consistently higher in rural than in urban areas (*u* = 41.0, *p* < 0.01), with the urban-to-rural ratio of ASMR rising from 0.90 in 2009 to 1.06 in 2018 before declining to 0.93 in 2021. The cause-specific mortality composition ratio showed a gradual increase in both areas, reaching 0.54% in urban and 0.48% in rural areas by 2021 (Table 1).

**Table 1:** CMR (per 100,000 population), ASMR (per 100,000 population), and cause-specific mortality composition ratios (%) for mental disorders among urban and rural residents in China, 2009–2021.

Year	Urban			Rural			Urban-to-rural ratio of ASMR
	CMR	ASMR	Cause-specific mortality composition ratio	CMR	ASMR	Cause-specific mortality composition ratio	
2009	3.60	3.86	0.58	3.08	4.28	0.47	0.90
2010	2.90	3.64	0.47	2.99	4.64	0.48	0.78
2011	2.47	2.62	0.40	3.15	4.15	0.49	0.63
2012	2.00	2.07	0.33	3.10	3.56	0.47	0.58
2013	2.86	3.10	0.46	2.72	3.27	0.41	0.95
2014	2.66	2.95	0.43	2.70	3.07	0.41	0.96
2015	2.79	3.09	0.45	2.83	3.21	0.43	0.96
2016	2.72	2.89	0.44	2.85	2.98	0.42	0.97
2017	2.71	2.86	0.44	2.78	2.87	0.41	1.00
2018	2.96	2.93	0.47	2.81	2.76	0.41	1.06
2019	3.10	3.02	0.49	2.86	2.96	0.41	1.02
2020	3.15	2.77	0.50	3.07	2.98	0.44	0.93
2021	3.45	2.77	0.54	3.54	2.99	0.48	0.93

#### 3.1.2 Mortality by age and sex

Across the study period, male ASMR was significantly higher than female ASMR in urban areas (*t* = 2.14, *p* = 0.043) and in rural areas (*t* = 2.72, *p* = 0.012). In contrast, CMR did not differ significantly between sexes in either area (Table 2). ASMR increased sharply with age. Mortality rates among children and adolescents aged (<20 years) remained below 0.5 per 100,000 throughout the study period. The highest rates were consistently observed in the oldest age groups (≥75 years), reaching 174.12 per 100,000 in rural males in 2009. A notable increase was observed in the 20–25 years age group from 2018 to 2021 in both urban and rural areas (Table 3).

**Table 2:** Gender-specific mortality rates (per 100,000 population) for mental disorders among urban and rural residents in China, 2009–2021.

Year	Urban males		Urban females		Rural males		Rural females	
	CMR	ASMR	CMR	ASMR	CMR	ASMR	CMR	ASMR
2009	3.35	4.04	3.85	3.57	2.71	7.42	3.46	4.15
2010	2.82	3.88	2.98	3.33	2.79	5.07	3.19	4.23
2011	2.31	2.74	2.63	2.45	2.91	4.41	3.41	3.83
2012	2.04	2.29	1.97	1.83	3.14	4.03	3.05	3.03
2013	2.85	3.40	2.88	2.75	2.68	3.56	2.76	2.92
2014	2.68	3.29	2.64	2.59	2.70	3.44	2.70	2.67
2015	2.73	3.34	2.86	2.80	2.66	3.39	3.01	2.96
2016	2.60	3.08	2.83	2.66	2.78	3.34	2.92	2.61
2017	2.60	3.08	2.83	2.63	2.74	3.24	2.82	2.48
2018	2.79	3.12	3.12	2.69	2.70	3.05	2.92	2.45
2019	2.91	3.18	3.30	2.82	2.72	3.18	2.99	2.69
2020	2.98	3.01	3.33	2.51	2.94	3.31	3.21	2.64
2021	3.07	2.87	3.84	2.64	3.29	3.26	3.81	2.71

**Table 3:** ASMR (per 100,000 population) for mental disorders among urban and rural residents in China, 2009–2021.

Age group	2009		2012		2015		2018		2021	
	Urban	Rural	Urban	Rural	Urban	Rural	Urban	Rural	Urban	Rural
<1	0	0.28	0	0	0.15	0.07	0.26	0.09	0	0
1 ~ <5	0.06	0	0	0	0.03	0.02	0.03	0.02	0.08	0.01
5 ~ <10	0.05	0	0	0	0.03	0.04	0.02	0.03	0.02	0.05
10 ~ <15	0	0.03	0.04	0.03	0.09	0.07	0.05	0.11	0.04	0.04
15 ~ <20	0.24	0.22	0.10	0.20	0.08	0.22	0.13	0.16	0.12	0.26
20 ~ <25	0.33	0.45	0.19	0.40	0.18	0.18	0.14	0.17	0.41	0.40
25 ~ <30	0.57	0.63	0.36	0.79	0.46	0.62	0.35	0.45	0.25	0.44
30 ~ <35	1.08	0.64	0.45	0.71	0.70	0.76	0.55	0.73	0.34	0.49
35 ~ <40	1.11	1.23	0.72	1.17	0.69	1.08	0.66	0.81	0.61	0.75
40 ~ <45	1.71	1.69	0.93	1.51	1.11	1.13	0.73	0.96	0.65	0.89
45 ~ <50	2.44	1.48	1.40	2.24	1.02	1.05	0.81	1.20	0.85	1.23
50 ~ <55	2.18	1.75	0.88	1.21	2.14	1.55	1.55	1.81	1.24	1.30
55 ~ <60	2.66	2.21	1.25	1.96	1.47	1.16	1.37	1.27	1.22	1.57
60 ~ <65	3.27	3.28	2.35	3.20	2.36	2.69	2.84	2.22	2.08	1.99
65 ~ <70	3.97	4.12	2.67	3.59	4.04	4.17	4.71	3.45	2.97	3.68
70 ~ <75	6.41	7.96	5.02	6.57	6.50	7.52	6.39	6.43	6.47	7.22
75 ~ <80	17.53	24.72	11.69	19.04	14.42	17.89	11.34	11.86	14.27	16.39
80 ~ <85	52.39	57.97	24.96	43.15	39.33	42.57	34.73	33.20	35.41	36.08
≥85	135.17	174.12	57.87	130.91	128.89	120.29	139.56	111.78	128.44	124.22

### 3.2 Joinpoint trend analysis

#### 3.2.1 Trends in CMR and ASMR by area

**Figure 1:**
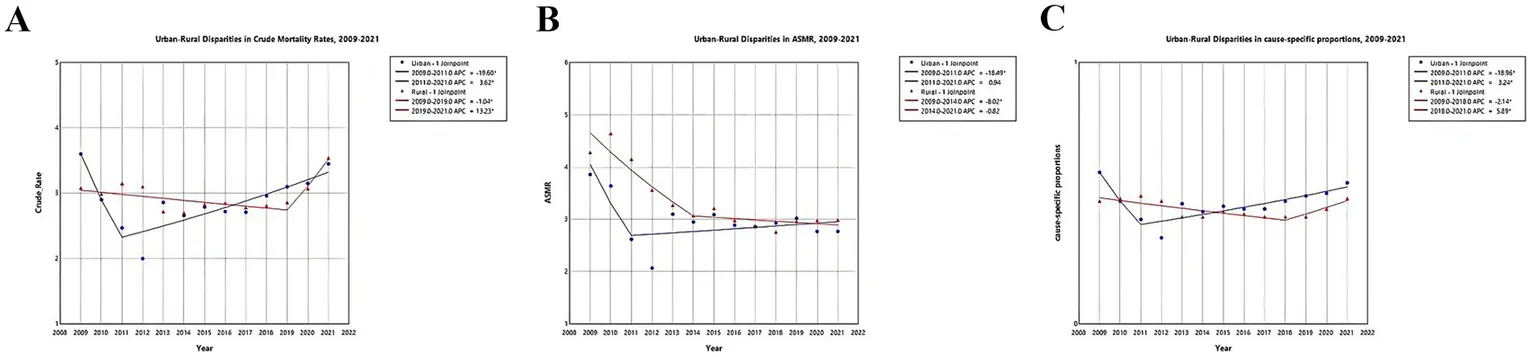
Trends in mortality rates and cause-specific composition ratio for mental disorders among urban and rural residents in China, 2009–2021. **(A)** Crude mortality rate (CMR, per 100,000 population); **(B)** Age-standardized mortality rate (ASMR, per 100,000 population); **(C)** Cause-specific mortality composition ratio (percentage of mental disorder deaths among all-cause deaths).

Joinpoint regression identified distinct temporal patterns. For urban residents, the CMR showed no significant overall trend (AAPC = −0.67%, *p* = 0.48), but a significant decline from 2009 to 2011 (APC = −19.60%, *p* < 0.01) followed by a significant increase from 2011 to 2021 (APC = 3.62%, *p* < 0.01). In contrast, rural CMR increased significantly overall (AAPC = 1.21%, *p* = 0.02), with a sharp rise after 2019 (APC = 13.23%, *p* < 0.01) following a period of decline from 2009 to 2019 (APC = −1.04%, *p* = 0.01; Figure 1A). The ASMR declined significantly only in rural areas (AAPC = 3.88%, *p* < 0.01; Figure 1B). For the cause-specific mortality composition ratio, urban areas showed a significant increase from 2011 to 2021 (APC = 3.24%, *p* < 0.01), and rural areas exhibited a sharp rise after 2018 (APC = 5.89%, *p* = 0.02; Figure 1C).

#### 3.2.2 Sex-specific trends

**Figure 2:**
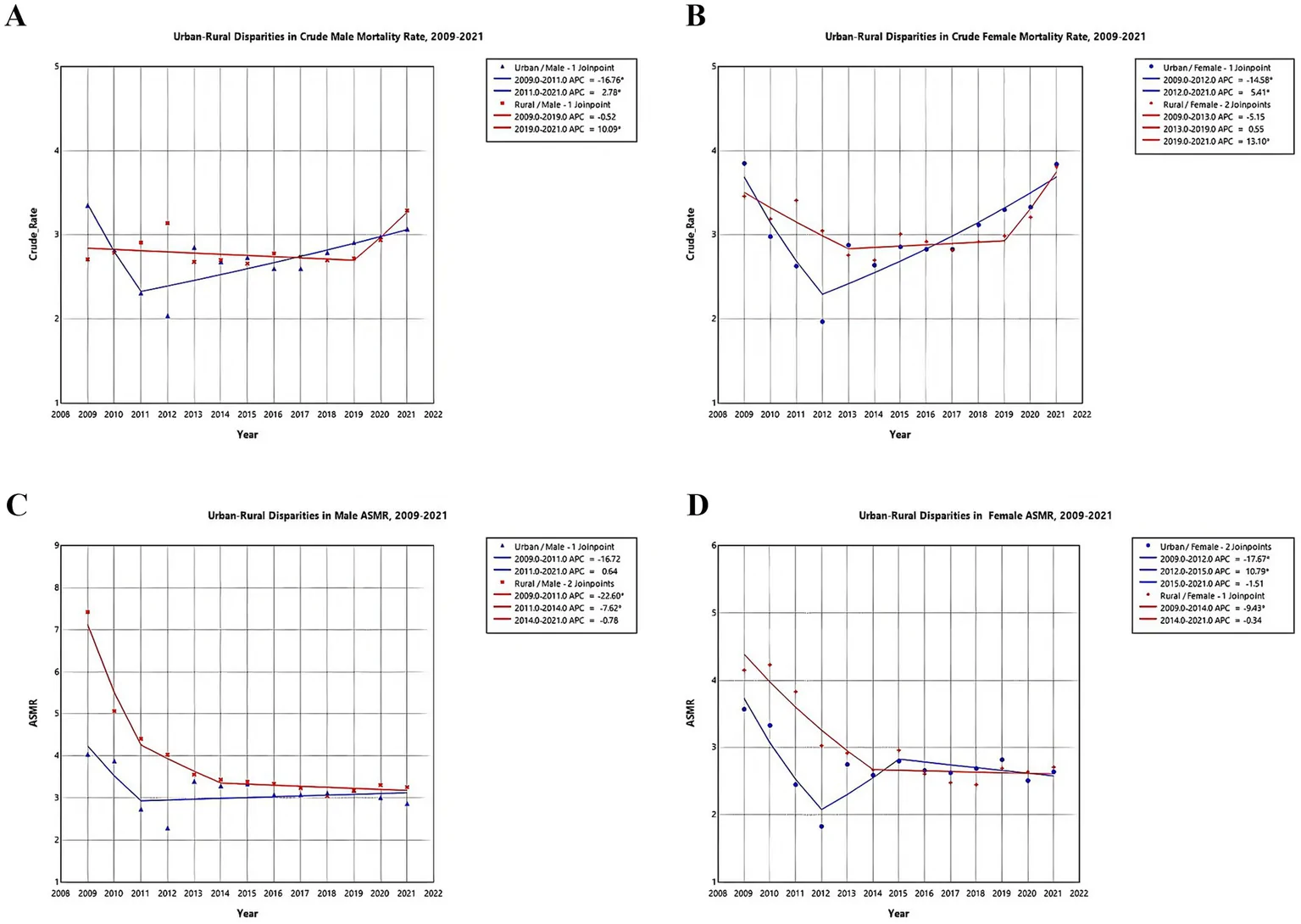
Trend change in gender-specific mortality rates for mental disorders among urban and rural residents in China, 2009–2021. **(A)** Crude mortality rate (CMR) for males; **(B)** CMR for females; **(C)** Age-standardized mortality rate (ASMR) for males; **(D)** ASMR for females.

In urban areas, male CMR declined significantly from 2009 to 2011 (APC = −16.76%, *p* < 0.01) and then increased significantly from 2011 to 2021 (APC = 2.78%, *p* < 0.01; Figure 2A). Urban female CMR showed a similar pattern: a decline from 2009 to 2012 (APC = 14.58%, *p* = 0.02) followed by an increase from 2012 to 2021 (APC = 5.41%, *p* < 0.01; Figure 2B). In rural areas, both sexes exhibited a sharp increase from 2019 to 2021 (male: APC = 10.09%, *p* < 0.01; female: APC = 13.10%, *p* < 0.01; Figure 2C). The ASMR for rural males declined more steeply (AAPC = −6.49%, *p* < 0.01) than for rural females (AAPC = −4.23%, *p* < 0.01; Figure 2D; Supplementary Table S1).

#### 3.2.3 Age group-specific trends

Among age groups (<20 years), Joinpoint models could not be fitted for most groups due to the high proportion of zero mortality values. For the 15 ~ <20 group, no significant temporal trend was detected in either urban (AAPC = −5.22%, *p* = 0.129) or rural areas (AAPC = 5.45%, *p* = 0.109). Most age groups (≥20 years) showed declining trends from 2009 to 2021, but the oldest age groups (≥75 years) consistently had the highest mortality rates. In urban areas, significant declines were observed in the 25 ~ 60 years groups. In rural areas, significant declines were found in the 35 ~ 50, 60 ~ 65, and ≥75 years groups. The 20 ~ <25 years age group showed an increase from 2018 to 2021 in both urban (APC = 33.01%, *p* = 0.12) and rural areas (APC = 23.11%, *p* = 0.12), but this trend did not reach statistical significance (Supplementary Table S2).

### 3.3 Panel regression analysis of influencing factors

#### 3.3.1 Stepwise regression results

The Durbin-Watson statistics ranged from 1.92 to 2.05, indicating no significant autocorrelation. The VIF for all variables in Model 4 were below 5, indicating no serious multicollinearity. And Model 4 included 14 covariates plus year fixed effects, corresponding to 27 estimated parameters.

The results showed that healthcare resource variables were significantly associated with lower ASMR in Model 1. After adding socioeconomic variables, income, insurance coverage, and urbanization showed protective associations in Model 2. Aging rate, incorporated in Model 3, became the strongest predictor (*β* = 0.791, *p* < 0.01), raising *r^2^* from 0.401 to 0.478. Model 4 further added mental disorder prevalence, suicide-specific mortality, smoking rate, and alcohol consumption. Aging rate remained the dominant factor (*β* = 0.650, *p* < 0.01), and adding these variables *r^2^* increased to 0.602. Several variables significant in earlier models became non-significant in Model 4, suggesting partial confounding by the newly added variables (Table 4).

**Table 4:** Regression analysis of factors influencing mortality rates from mental disorders among urban and rural residents in China.

Variable	Model 1	Model 2	Model 3	Model 4
	*β (SE)*	*β (SE)*	*β (SE)*	*β (SE)*
Psychiatric bed density	−0.013 (0.001)^*^	−0.012 (0.001)^*^	−0.014 (0.001)^*^	−0.010 (0.001)^*^
Psychiatrist density	−0.008 (0.004)^*^	−0.007 (0.004)	−0.009 (0.004)^*^	−0.006 (0.004)
Psychotherapist density	−0.020 (0.009)^*^	−0.019 (0.009)	−0.021 (0.009)^*^	−0.015 (0.008)^*^
Community health center density	−0.002 (0.001)^*^	−0.002 (0.001)^*^	−0.002 (0.001)^*^	−0.001 (0.001)
Psychiatrist workload	−0.001 (0.001)^*^	−0.001 (0.001)^*^	−0.001 (0.001)^*^	−0.001 (0.001)^*^
Per capita disposable income	–	−0.400 (0.190)^*^	−0.423 (0.185)^*^	−0.300 (0.1701)
Basic medical insurance coverage rate	–	−0.045 (0.018)^*^	−0.050 (0.018)^*^	−0.035 (0.016)^*^
Urbanization rate	–	−0.030 (0.015)^*^	−0.035 (0.015)^*^	−0.020 (0.014)
Aging rate	–	–	0.791 (0.085)^*^	0.650 (0.080)^*^
Mental disorder prevalence	–	–	–	0.420 (0.095)^*^
Suicide-specific mortality	–	–	–	0.350 (0.070)^*^
Adult smoking rate	–	–	–	0.180 (0.050)^*^
Alcohol consumption	–	–	–	0.120 (0.045)^*^
Urban (ref: rural)	−1.019 (0.528)	−0.980 (0.520)	−1.019 (0.528)	−0.800 (0.500)
*r^2^*	0.324	0.401	0.478	0.602
*f*	28.6^*^	31.2^*^	38.5^*^	45.2^*^
Durbin-Watson	1.92	1.98	2.01	2.05

Dashes (–) indicate variables not included in the model. *p < 0.05.

Models 1–4 re-estimated with data from 2009 to 2019 only yielded consistent results, with aging rate remaining the strongest predictor (*β* = 0.780, *p* < 0.01; Supplementary Table S3). A second sensitivity analysis excluding dementia related deaths was conducted for the 2013–2021 period. The aging coefficient decreased from 0.617 to 0.435 (*p* < 0.01), and the overall pattern of associations for other variables was comparable (Supplementary Table S4).

#### 3.3.2 Variable importance ranking

Standardized coefficient ranking based on Model 4 showed that aging rate had the largest standardized coefficient (*β* = 0.352), confirming its dominant role even after controlling for four previously omitted confounders. Suicide-specific mortality ranked second (*β* = 0.217), followed by mental disorder prevalence (*β* = 0.191) and adult smoking rate (*β* = 0.156). Among healthcare resource variables, psychiatrist workload and psychiatric bed density contributed most strongly, while psychotherapist density and psychiatrist density showed smaller and non-significant standardized associations. Per capita disposable income, urbanization rate, and community health center density were not statistically significant in Model 4 (Supplementary Table S5).

#### 3.3.3 Robustness to omitted variable bias

The results showed that mental disorder prevalence, suicide-specific mortality, smoking rate, and alcohol consumption remained highly robust after adjusting for unmeasured confounding, with adjusted coefficients ( $\beta^{*}$ ) of 1.032 , 0.860 , 0.442 , and 0.295 , respectively, under the assumption of δ=1 . Among the core variables, aging rate was the most sensitive ( δ=0.316 ), indicating that unmeasured confounders would need to be approximately one-third as important as the observed covariates to fully eliminate the aging mortality association. Urban–rural residence showed similar sensitivity ( δ=0.399 ). Healthcare resource and socioeconomic variables were generally robust, with required δ ranging from 0.583 to infinity. Overall, the protective associations of psychiatric resources and socioeconomic development appear robust to plausible levels of omitted variable bias, whereas the aging–mortality association is moderately sensitive (Table 5).

**Table 5:** Oster (2019) bounds analysis for omitted variable bias.

Variable	$\beta^{*}(\delta =1)$	required δ	Robustness
Psychiatric bed density	−0.004	0.583	Moderately robust
Psychiatrist density	−0.002	0.728	Moderately robust
Psychotherapist density	−0.006	0.583	Moderately robust
Community health center density†	0.0005	–	(n.s.)
Psychiatrist workload	−0.001	∞	Very robust
Per capita disposable income	−0.121	0.597	Moderately robust
Basic medical insurance coverage	−0.013	0.624	Moderately robust
Urbanization rate^†^	0.002	–	(n.s.)
Aging rate	0.445	0.316	Sensitive
Mental disorder prevalence^‡^	1.032	–	Very robust
Suicide-specific mortality^‡^	0.86	–	Very robust
Adult smoking rate^‡^	0.442	–	Very robust
Alcohol consumption^‡^	0.295	–	Very robust
Urban (ref: rural)	−0.481	0.399	Sensitive

$\beta^{*}$ the adjusted coefficient assuming δ=1 ; δ indicates the required value to reduce $\beta_{f}$ to zero; + indicates not significant in Model 4; ^†^ Indicates not included in Model 3; (n.s.) indicates not significant in Model 4. *p <0.05.

### 3.4 Forecast of mortality trends

#### 3.4.1 Model comparison

**Figure 3:**
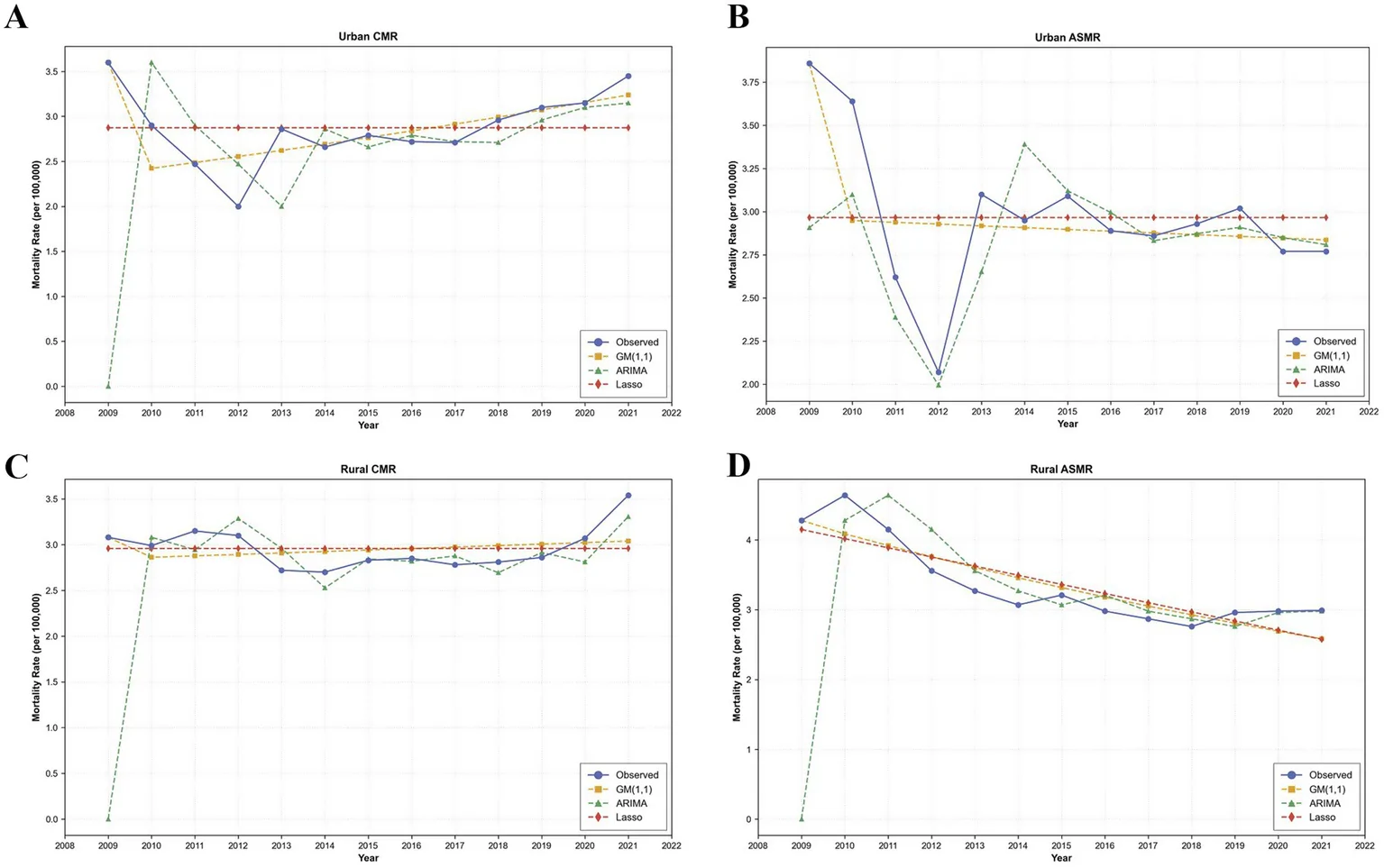
Comparison of model fitting for GM (1,1), ARIMA, and Lasso regression across four mortality series, 2009–2021. **(A)** Urban crude mortality rate (CMR); **(B)** Urban age-standardized mortality rate (ASMR); **(C)** Rural CMR; **(D)** Rural ASMR.

The result showed that the forecasting performance of the three models on the 2018–2019 validation set. GM (1,1) achieved the lowest RMSE for urban CMR (0.268), followed by Lasso (0.293) and ARIMA (0.328). For urban ASMR, Lasso performed best (RMSE = 0.056), followed by ARIMA (0.124) and GM (1,1) (0.155). ARIMA was optimal for rural CMR (RMSE = 0.037), outperforming Lasso (0.150) and GM (1,1) (0.173), and for rural ASMR (RMSE = 0.101). The automatically selected ARIMA orders were (0,1,0), a random walk without drift, for urban CMR, urban ASMR, and rural ASMR, and (0,0,1), a simple moving average, for rural CMR (Table 6; Figure 3).

**Table 6:** Comparison of forecasting model performance on the validation set.

Series	GM (1,1)		ARIMA			Lasso		
	RMSE	MAE	RMSE	MAE	Order	RMSE	MAE	Alpha
Urban CMR	0.268	0.262	0.328	0.320	(0, 1, 0)	0.293	0.284	0.546
Urban ASMR	0.154	0.146	0.124	0.115	(0, 1, 0)	0.056	0.045	0.886
Rural CMR	0.173	0.167	0.037	0.028	(0, 0, 1)	0.150	0.143	0.030
Rural ASMR	0.488	0.451	0.101	0.100	(0, 1, 0)	0.496	0.452	0.048

RMSE, root mean square error; MAE, mean absolute error. ARIMA Order: (p,d,q) parameters selected by minimum AIC. Lasso alpha was selected by leave-one-out cross-validation.

Based on the criteria, GM (1,1) was selected to generate the baseline projections. Although ARIMA achieved lower validation errors for three of the four series, its random walk and simple moving average specifications produce forecasts that converge to constant values, offering no directional guidance for assessing whether current trends are likely to continue. Lasso, limited to a single predictor linear structure, does not capture the non-linear trends present in several of the series. By contrast, GM (1,1) generates directional projections that are broadly aligned with the trend breaks identified by Joinpoint analysis, satisfying both criteria. The ARIMA and Lasso forecasts, which reflect different modeling assumptions, were retained for constructing the optimistic and pessimistic scenario bounds. ARIMA’s conservative projections, which assume no directional change, provide a lower bound for the uncertainty range, while Lasso’s linear extrapolations offer an intermediate trajectory between the two.

#### 3.4.2 Scenario projections

**Figure 4:**
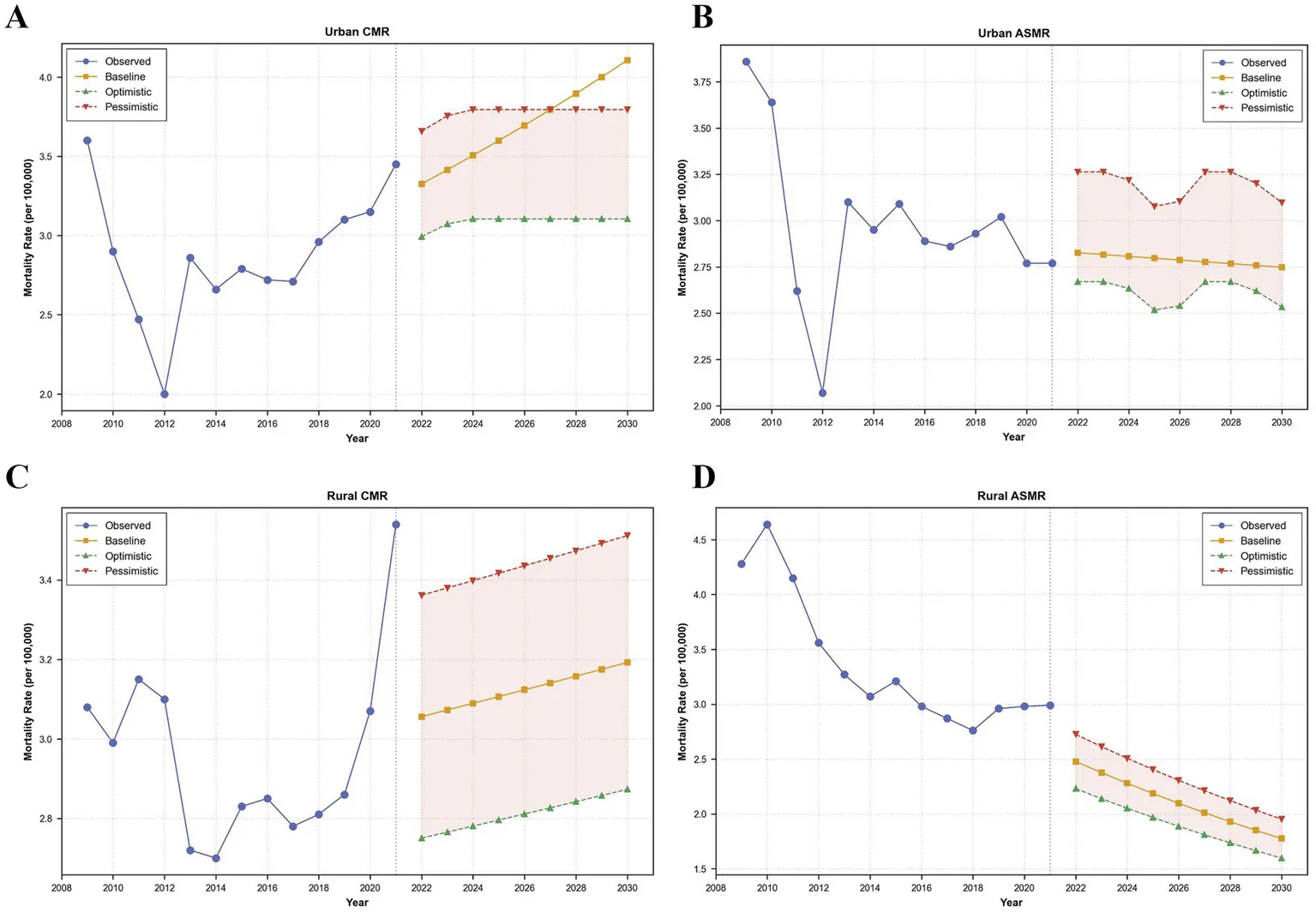
Scenario projections of mental disorder mortality to 2030. **(A)** Urban crude mortality rate (CMR); **(B)** Urban age-standardized mortality rate (ASMR); **(C)** Rural CMR; **(D)** Rural ASMR.

GM (1,1) baseline projections indicated that urban CMR would increase from 3.33 per 100,000 in 2022 to 4.11 per 100,000 in 2030, while urban ASMR would decline slightly from 2.83 to 2.75 per 100,000 over the same period. Rural CMR was projected to increase modestly from 3.06 to 3.19 per 100,000, and rural ASMR was projected to decline from 2.48 to 1.77 per 100,000. Under the optimistic scenario, defined as 90% of the three-model median forecast, the 2030 projections were 3.11, 2.53, 2.87, and 1.60 per 100,000 for urban CMR, urban ASMR, rural CMR, and rural ASMR, respectively. Under the pessimistic scenario, the corresponding projections were 3.80, 3.10, 3.51, and 1.95 per 100,000 (Figure 4).

## 4 Discussion

This study revealed divergent mortality trends between urban and rural in China. Urban CMR showed no significant overall trend across the study period, declining sharply during 2009–2011 and then rising gradually through 2021. This early decline coincided with the implementation of the Severe Mental Disorder Management and Treatment Program and the integration of mental health into primary care. Urban areas, with better infrastructure and a higher concentration of specialized personnel, may have benefited more rapidly from these policy initiatives. Notably, the urban-to-rural ratio of ASMR exceeded 1.0 in 2018 and 2019, suggesting that urban areas experienced a relatively higher ASMR during this period. This may be linked to emerging specific urban stressors such as social alienation, loneliness, and cost of living pressures, which have been linked to increased suicide and substance related deaths in urban populations (22, 23). And national surveys also revealed that perceived social isolation increased significantly from 2010 to 2020 in urban populations and was independently associated with elevated psychological distress (24).

In contrast, rural CMR increased significantly over the study period, with a sharp rise during 2019–2021. The surge may reflect multiple contributing factors. Among them, two explanations deserve consideration. One is the disruption caused by the COVID-19 pandemic. According to statistics, over 80% medical institutions experienced service disruptions during the early pandemic period, with rural primary care facilities facing the most severe shortages of medications and personnel (25). The other points to structural forces that predate the pandemic. Sustained rural-to-urban migration of working-age adults has left behind an increasingly older population with weakened family support networks (26). The progressive acceleration of rural CMR increases through 2021, rather than a spike followed by attenuation, lends support to the structural interpretation, although both mechanisms likely interacted. The regression results further support this interpretation. Urbanization rate was negatively associated with ASMR, indicating that areas with higher levels of urbanization tended to have lower mental disorder mortality. Compounding these challenges, stigma toward mental disorders remains higher in rural communities. And over 40% of Chinese rural residents endorsed beliefs that mental illness reflects personal weakness, compared with approximately 25% in urban areas, potentially delaying treatment and worsening outcomes (27).

Age-specific mortality exhibited a clear gradient with advancing age. Mortality rates among those aged under 20 years remained below 0.5 per 100,000 throughout the study period, and no significant temporal trends were detected in these groups. However, the 20 ~ <25 years age group showed an increase from 2018 to 2021 in both urban and rural areas. Although this trend did not reach statistical significance (*p* = 0.12), the observed increase warrants attention. National surveys have documented rising depressive symptoms among college students, with the prevalence increasing from 21.6% in 2017 to 27.8% in 2020, with academic pressure, employment uncertainty, and reduced social interaction identified as contributing factors, (28), and the 2021 China National Mental Health Survey identified young adults aged 18–24 years as the age group with the highest level of psychological distress (29). The result suggest that the transition to young adulthood could be a period of heightened vulnerability, and the mortality increase observed here may be an early signal that warrants continued monitoring.

Consistent with previous studies (30, 31), ASMR was significantly higher in males than in females in both urban and rural areas. This disparity may be attributed to traditional gender norms that discourage emotional expression and help-seeking behavior in men, leading to delayed diagnosis and treatment. And higher rates of substance use disorders and impulse control disorders among males, which often co-occur with cardiovascular and other somatic conditions, collectively increasing mortality risk (32).

The stepwise panel regression identified aging rate as the strongest factor associated with mental disorder mortality. This association persisted after controlling for additional confounders in Model 4, suggesting that population aging is not simply a proxy for other measured factors. Among healthcare resource variables, higher densities of psychiatric beds and psychotherapists were independently associated with lower ASMR, whereas psychiatrist density and community health center density were not significant. This pattern suggests that resource quantity alone may be insufficient without corresponding improvements in service quality and accessibility (33). The negative association between psychiatrist workload and mortality likely reflects a compensatory mechanism in resource-limited settings and should not be interpreted as evidence that higher workload is beneficial (34). Urban–rural residence was not significant in the full model, implying that the observed urban–rural mortality gap is largely explained by disparities in healthcare resources, socioeconomic conditions, and population aging.

And several sensitivity analyses were conducted to assess the robustness of these findings. Excluding the pandemic years did not alter the overall pattern of associations. A further analysis removing dementia-related deaths showed that the aging coefficient decreased but remained significant, indicating that the aging-mortality association is not solely attributable to dementia. Other age-related factors, including multimorbidity, functional decline, and reduced social support, may play additional roles. These results, together with the Oster boundary analysis, reinforce the conclusion that the protective associations of psychiatric resources are generally robust to plausible sources of bias.

The scenario projections indicated that even under the optimistic scenario, rural CMR remained above pre-2019 levels, while the pessimistic scenario approached the elevated values observed in 2020–2021. For urban CMR, the baseline and pessimistic scenarios both pointed to continued growth, and even the optimistic scenario did not suggest a return to earlier, lower levels. This pattern aligns with the structural break in rural mortality detected by Joinpoint analysis, suggesting that the post-2019 rural surge may have lasting effects that are unlikely to be quickly reversed. These results imply that urban and rural areas may face different challenges in the coming decade, with urban mortality likely to continue rising and rural mortality remaining above its pre-pandemic baseline under most plausible assumptions. The gap between the optimistic and pessimistic scenarios highlights the sensitivity of future mortality to policy choices and demographic trends. The overall direction of these projections, continued urban growth and persistently elevated rural mortality, remained consistent across the range of scenarios examined. These projections should be interpreted as conditional rather than unconditional forecasts.

Based on the findings, the following recommendations are proposed. First, given the strong association between population aging and mental disorder mortality, geriatric mental health services need to be expanded. This includes integrating mental health care into routine primary care for older adults and developing community-based programs for patients with both mental disorders and chronic physical conditions (35). Second, the observed increase in mortality among young adults aged 20–25 years, suggests that mental health interventions should begin earlier in life through school-based mental health literacy programs, university counseling services, and workplace mental health initiatives. Third, urban–rural disparities in mortality require differentiated resource allocation. In rural areas, strengthening the primary-care mental health workforce and expanding community-based service networks should be priorities (36). In urban areas, integrating mental health services into workplace settings and expanding digital mental health platforms may help reach populations who do not seek traditional care.

## 5 Conclusion

This study analyzed mortality trends for mental disorders among Chinese urban and rural residents from 2009 to 2021. Rural CMR increased significantly over the study period, with a sharp rise during 2019–2021, while urban CMR remained relatively stable. Age-specific mortality rose steeply with advancing age, with the heaviest burden consistently falling on the oldest age groups. Mortality rates among those aged under 20 years were extremely low throughout the study period, and the 20–25 years age group showed an increase from 2018 to 2021, although this trend did not reach statistical significance. Aging rate showed the strongest association with mental disorder mortality, and this association persisted after controlling for additional confounders. Psychiatric resources and socioeconomic development were associated with lower mortality. Scenario projections suggested continued differences between urban and rural trends, with the gap between optimistic and pessimistic scenarios reflecting the sensitivity of future trajectories to policy choices. The findings point to the need for strengthening geriatric mental health services, extending interventions across the life course, and addressing urban–rural disparities through differentiated resource allocation.

## 6 Limitations

Several limitations should be acknowledged. First, due to the availability of official yearbook data, this study only covers the period 2009–2021. The absence of post-2021 data limits our ability to assess whether the observed trends have persisted or reversed. And dementia-specific mortality data were available only from 2013 onward, restricting the sensitivity analysis excluding dementia-related deaths to a shorter time frame. Second, the absence of data on specific mental disorder subtypes and regional variations precludes disaggregated analysis that could inform more targeted prevention strategies. Third, the panel data include only two cross-sectional units observed over 13 years. Given the limited sample size, the relatively large number of covariates in the fully adjusted model may lead to instability in the adjusted estimates. Fourth, the ecological panel design precludes causal inference. Unmeasured confounders, including treatment adherence and individual-level lifestyle factors, may bias the estimated associations.

## 7 Future research directions

Several directions for future research emerge from this study. First, extended time series analyses incorporating post-2021 data are needed to determine whether the sharp increase in rural mortality during 2019–2021 was sustained or transient. Second, analyses of mortality by specific mental disorder subtypes would help clarify whether the observed trends are driven by particular diagnostic categories. Third, studies at the provincial or regional level would help identify geographic disparities that the binary urban–rural classification cannot capture.

## Data Availability

Publicly available datasets were analyzed in this study. This data can be found at: http://www.stats.gov.cn and http://www.nhc.gov.cn.
