# Supplementary material for: Mortality trends and influencing factors of mental disorders among urban and rural residents in China between 2009 and 2021: a longitudinal study with projections to 2030
Source: Front Public Health. 2026 Sep 3;14:1851033. doi: 10.3389/fpubh.2026.1851033 (PMC13581816; doi:10.3389/fpubh.2026.1851033)
Supplement: Supplementary file 1 [file Data_Sheet_1.ZIP › Supplementary Table/Supplementary Table S1. Trends in mortality rates for mental disorders among urban and rural residents in China, 2009 - 2021.docx]

**Supplementary table S1.** Trends in mortality rates for mental disorders among urban and rural residents in China, 2009 - 2021

| Indicator | Area | Sex | AAPC  (95%CI) | Trend 1 | | Trend 2 | | Trend 3 | |
| --- | --- | --- | --- | --- | --- | --- | --- | --- | --- |
|  |  |  |  | Period | APC  (95%CI) | Period | APC  (95%CI) | Period | APC  (95%CI) |
| CMR | Urban | Overall | -0.67  (-4.85, 3.68) | 2009-2011 | -19.60*  (-39.37, 6.62) | 2011-2021 | 3.62*  (1.36, 5.92) | — | — |
|  |  | Male | -0.77 (-2.07, 1.21) | 2009-2011 | -16.76* (-23.41, -3.75) | 2011-2021 | 2.78* (1.33, 5.96) | — | — |
|  |  | Female | 0.01 (-1.68, 2.30) | 2009-2012 | -14.58* (-26.87, -4.02) | 2012-2021 | 5.41* (3.16,10.33) | — | — |
|  | Rural | Overall | 1.21*  (-0.95, 3.41) | 2009-2019 | -1.04*  (-2.13, 0.06) | 2019-2021 | 13.23*  (-1.75, 30.50) | — | — |
|  |  | Male | 1.18 (-0.03, 2.12) | 2009-2019 | -0.52 (-3.06, 0.31) | 2019-2021 | 10.09 (1.68,16.84) | — | — |
|  |  | Female | 0.56 (-0.92, 1.74) | 2009-2013 | -5.15 (-11.85, 1.86) | 2013-2019 | 0.55 (-4.72, 4.57) | 2019-2021 | 13.10 (2.36, 19.42) |
| ASMR | Urban | Overall | -2.59  (-8.16, 3.32) | 2009-2011 | -18.49*  (-44.66, -20.07) | 2011-2021 | 0.94  (-2.06, 4.03) | — | — |
|  |  | Male | -2.49 (-4.51, 0.90) | 2009-2011 | -16.72 (-27.09, 0.94) | 2011-2021 | 0.64  (-12.74,14.71) | — | — |
|  |  | Female | -3.02* (-4.83, -1.58) | 2009-2012 | -17.67* (-28.32, -10.88) | 2012-2015 | 10.79* (2.33, 17.23) | 2015-2021 | -1.51 (-10.57, 1.39) |
|  | Rural | Overall | -3.88*  (-5.79, -1.94) | 2009-2014 | -8.02*  (-11.91, -3.95) | 2014-2021 | -0.82  (-3.35, 1.78) | — | — |
|  |  | Male | -6.49* (-7.13, -5.53) | 2009-2011 | -22.60* (-26.91, -15.89) | 2011-2014 | -7.62* (-10.18, -0.16) | 2014-2021 | -0.78 (-3.52, 3.39) |
|  |  | Female | -4.23* (-5.94, -2.28) | 2009-2014 | -9.43* (-21.74, -4.15) | 2014-2021 | -0.34 (-3.83, 12.68) | — | — |
| Cause-  Specific mortality composition ratio | Urban | — | -0.84  (-2.14, 1.37) | 2009-2011 | -18.96*  (-25.35, -4.41) | 2011-2021 | 3.24*  (1.63, 6.65) | — | — |
|  | Rural | — | -0.19  (-1.28, 0.55) | 2009-2018 | -2.14*  (-4.69, -1.13) | 2018-2021 | 5.89*  (0.64, 13.00） | — | — |
| *Notes:* Dashes (—) indicate no further segments identified. **P*<0.05 | | | | | | | | | |
