# Supplementary material for: Mortality trends and influencing factors of mental disorders among urban and rural residents in China between 2009 and 2021: a longitudinal study with projections to 2030
Source: Front Public Health. 2026 Sep 3;14:1851033. doi: 10.3389/fpubh.2026.1851033 (PMC13581816; doi:10.3389/fpubh.2026.1851033)
Supplement: Supplementary file 1 [file Data_Sheet_1.ZIP › Supplementary Table/Supplementary Table S2. Trends in age‐specific mortality rates for mental disorders among urban and rural residents in China, 2009 - 2021.docx]

**Supplementary table S2.** Trends in age‑specific mortality rates for mental disorders among urban and rural residents in China, 2009 - 2021

| Area | Age group | AAPC (95%CI) | Trend 1 | | Trend 2 | | Trend 3 | |
| --- | --- | --- | --- | --- | --- | --- | --- | --- |
|  |  |  | Period | APC  (95%CI) | Period | APC  (95%CI) | Period | APC  (95%CI) |
| Urban | < 1 | — | — | — | — | — | — | — |
|  | 1～< 5 | — | — | — | — | — | — | — |
|  | 5～< 10 | — | — | — | — | — | — | — |
|  | 10～<15 | — | — | — | — | — | — | — |
|  | 15～< 20 | -5.22  (-11.80, 1.85) | 2009-2021 | -5.22  (-11.80, 1.85) | — | — | — | — |
|  | 20～< 25 | 2.03  (-4.85, 6.70) | 2009-2018 | -6.61  (-31.36, 1.32) | 2018-2021 | 33.01  (-1.52, 91.00) | — | — |
|  | 25～< 30 | -6.04*  (-11.27, -1.00) | 2009-2014 | 3.40  (-6.92, 45.95) | 2014-2021 | -12.25* (-37.11, -5.96) | — | — |
|  | 30～< 35 | -6.90*  (-10.67, -2.80) | 2009-2021 | -6.90*  (-10.67, -2.80) | — | — | — | — |
|  | 35～< 40 | -4.55*  (-7.62, -1.35) | 2009-2021 | -4.55*  (-7.62, -1.35) | — | — | — | — |
|  | 40～< 45 | -6.41*  (-9.96, -2.73) | 2009-2021 | -6.41*  (-9.96, -2.73) | — | — | — | — |
|  | 45～< 50 | -6.17*  (-9.04, -2.99) | 2009-2014 | -14.50*  (-32.32, -6.74) | 2014-2021 | 0.27  (-5.02, 20.61) | — | — |
|  | 50～< 55 | -4.41*  (-6.70, -2.18) | 2009-2012 | -21.33*  (-36.72,-11.80) | 2012-2015 | 28.02*  (11.66, 39.71) | 2015-2021 | -8.95*  (-15.28,-5.23) |
|  | 55～< 60 | -4.55*  (-8.24, -0.64) | 2009-2021 | -4.55*  (-8.24, -0.64) | — | — | — | — |
|  | 60～< 65 | -2.86  (-6.08, 0.56) | 2009-2021 | -2.86  (-6.08, 0.56) | — | — | — | — |
|  | 65～< 70 | 0.04  (-4.13, 4.50) | 2009-2021 | 0.04  (-4.13, 4.50) | — | — | — | — |
|  | 70～< 75 | 0.19  (-1.44, 1.81) | 2009-2021 | 0.19  (-1.44, 1.81) | — | — | — | — |
|  | 75～< 80 | -1.48  (-3.97, 0.47) | 2009-2017 | -5.71*  (-16.11, -2.65) | 2017-2021 | 7.55  (-1.05, 27.56) | — | — |
|  | 80～< 85 | -3.63*  (-5.47, -2.08) | 2009-2012 | -18.65*  (-30.00,-11.29) | 2012-2015 | 11.16*  (2.57, 18.40) | 2015-2021 | -2.33  (-11.36, 0.28) |
|  | ≥85 | -1.25  (-5.35, 2.45) | 2009-2012 | -23.04*  (-43.58, -6.23) | 2012-2015 | 26.38*  (8.40, 45.39) | 2015-2021 | -1.13  (-20.71, 4.09) |
| Rural | < 1 | — | — | — | — | — | — | — |
|  | 1～< 5 | — | — | — | — | — | — | — |
|  | 5～< 10 | — | — | — | — | — | — | — |
|  | 10～<15 | — | — | — | — | — | — | — |

**Table S2 (continued)**

| Area | Age group | AAPC (95%CI) | Trend 1 | | Trend 2 | | Trend 3 | |
| --- | --- | --- | --- | --- | --- | --- | --- | --- |
|  |  |  | Period | APC  (95%CI) | Period | APC  (95%CI) | Period | APC  (95%CI) |
|  | 15～< 20 | 5.45  (-1.38, 12.76) | 2009-2021 | 5.45  (-1.38, 12.76) | — | — | — | — |
|  | 20～< 25 | -1.26  (-6.86, 2.78) | 2009-2018 | -8.26*  (-29.49, -1.62) | 2018-2021 | 23.11  (-3.67, 68.83) | — | — |
|  | 25～< 30 | -4.29  (-9.27, 1.07) | 2009-2021 | -4.29  (-9.27, 1.07) | — | — | — | — |
|  | 30～< 35 | -0.83  (-5.93, 4.44) | 2009-2014 | 11.82*  (1.35, 54.08) | 2014-2021 | -8.98*  (-28.78, -3.38) | — | — |
|  | 35～< 40 | -4.54*  (-7.08, -1.94) | 2009-2021 | -4.54*  (-7.08, -1.94) | — | — | — | — |
|  | 40～< 45 | -4.27*  (-5.95, -2.61) | 2009-2021 | -4.27*  (-5.95, -2.61) | — | — | — | — |
|  | 45～< 50 | -2.63*  (-5.14, -0.10) | 2009-2021 | -2.63*  (-5.14, -0.10) | — | — | — | — |
|  | 50～< 55 | -1.89  (-4.85, 1.14) | 2009-2021 | -1.89  (-4.85, 1.14) | — | — | — | — |
|  | 55～< 60 | -1.97  (-4.47, 0.66) | 2009-2011 | 10.59  (-11.21, 31.50) | 2011-2014 | -20.81  (-26.80, 7.71) | 2014-2021 | 3.78  (-4.41, 12.45) |
|  | 60～< 65 | -4.61*  (-6.94, -2.24) | 2009-2021 | -4.61*  (-6.94, -2.24) | — | — | — | — |
|  | 65～< 70 | -1.77  (-4.32, 0.83) | 2009-2021 | -1.77  (-4.32, 0.83) | — | — | — | — |
|  | 70～< 75 | -1.07  (-2.75, 0.69) | 2009-2021 | -1.07  (-2.75, 0.69) | — | — | — | — |
|  | 75～< 80 | -3.06*  (-5.35, -1.31) | 2009-2017 | -7.83*  (-15.40, -5.16) | 2017-2021 | 7.21  (-1.20, 25.68) | — | — |
|  | 80～< 85 | -3.70*  (-5.55, -2.22) | 2009-2017 | -7.12*  (-14.75, -4.95) | 2017-2021 | 3.53  (-2.80, 17.04) | — | — |
|  | ≥85 | -4.99*  (-7.39, -2.15) | 2009-2013 | -16.13*  (-30.60, -7.48) | 2013-2021 | 1.13  (-2.28, 10.89) | — | — |
| *Notes:* Dashes (—) indicate no further segments identified. **P*<0.05 | | | | | | | | |
