# Supplementary material for: Mortality trends and influencing factors of mental disorders among urban and rural residents in China between 2009 and 2021: a longitudinal study with projections to 2030
Source: Front Public Health. 2026 Sep 3;14:1851033. doi: 10.3389/fpubh.2026.1851033 (PMC13581816; doi:10.3389/fpubh.2026.1851033)
Supplement: Supplementary file 1 [file Data_Sheet_1.ZIP › Supplementary Table/Supplementary Table S3. Sensitivity analysis excluding the pandemic years.docx]

**Supplementary table S3.** Sensitivity analysis excluding the pandemic years

| Variable | Model 1 | Model 2 | Model 3 | Model 4 |
| --- | --- | --- | --- | --- |
|  | *β (SE)* | *β (SE)* | *β (SE)* | *β (SE)* |
| Psychiatric bed density | –0.012 (0.001)* | –0.011 (0.001)* | –0.013 (0.001)* | –0.010 (0.001)* |
| Psychiatrist density | –0.007 (0.004) | –0.006 (0.004) | –0.008 (0.004)* | –0.006 (0.004) |
| Psychotherapist density | –0.018 (0.009)* | –0.017 (0.009) | –0.020 (0.009)* | –0.013 (0.009) |
| Community health center density | –0.002 (0.001)* | –0.002 (0.001)* | –0.002 (0.001)* | –0.001 (0.001) |
| Psychiatrist workload | –0.001 (0.001)* | –0.001 (0.001)* | –0.001 (0.001)* | –0.001 (0.001)* |
| Per capita disposable income | — | –0.380 (0.190)* | –0.402 (0.187)* | –0.290 (0.176) |
| Basic medical insurance coverage | — | –0.042 (0.018)* | –0.048 (0.018)* | –0.033 (0.017)* |
| Urbanization rate | — | –0.028 (0.015) | –0.033 (0.015)* | –0.019 (0.014) |
| Aging rate | — | — | 0.780 (0.085)* | 0.645 (0.081)* |
| Mental disorder prevalence | — | — | — | 0.410 (0.095)* |
| Suicide‑specific mortality | — | — | — | 0.340 (0.071)* |
| Adult smoking rate | — | — | — | 0.172 (0.052)* |
| Alcohol consumption | — | — | — | 0.114 (0.046)* |
| Urban (ref: rural) | –1.019 (0.528) | –0.965 (0.522) | –1.019 (0.526) | –0.790 (0.504) |
| *R²* | 0.31 | 0.388 | 0.465 | 0.588 |
| *F* | 26.4* | 29.5* | 36.2* | 44.0* |
| Durbin‑Watson | 1.95 | 2 | 2.03 | 2.06 |
| *Notes:* Dashes (—) indicate variables not included in the model. **P*<0.05. | | | | |
