# Supplementary material for: Mortality trends and influencing factors of mental disorders among urban and rural residents in China between 2009 and 2021: a longitudinal study with projections to 2030
Source: Front Public Health. 2026 Sep 3;14:1851033. doi: 10.3389/fpubh.2026.1851033 (PMC13581816; doi:10.3389/fpubh.2026.1851033)
Supplement: Supplementary file 1 [file Data_Sheet_1.ZIP › Supplementary Table/Supplementary table S4. Sensitivity analysis excluding dementia deaths.docx]

**Supplementary table S4.** Sensitivity analysis excluding dementia deaths (Model 4)

| Variable | Total ASMR | Non-dementia ASMR |
| --- | --- | --- |
|  | *β (SE)* | *β (SE)* |
| Psychiatric bed density | –0.010 (0.001)* | –0.009 (0.001)* |
| Psychiatrist density | –0.006 (0.004) | –0.006 (0.004) |
| Psychotherapist density | –0.014 (0.010) | –0.013 (0.009) |
| Community health center density | –0.001 (0.001) | –0.001 (0.001) |
| Psychiatrist workload | –0.001 (0.001)* | –0.001 (0.001)* |
| Per capita disposable income | –0.287 (0.176) | –0.275 (0.172) |
| Basic medical insurance coverage | –0.032 (0.018)* | –0.028 (0.017) |
| Urbanization rate | –0.018 (0.014) | –0.018 (0.014) |
| Aging rate | 0.617 (0.086)* | 0.435 (0.083)* |
| Mental disorder prevalence | 0.402 (0.099)* | 0.385 (0.095)* |
| Suicide‑specific mortality | 0.337 (0.074)* | 0.322 (0.072)* |
| Adult smoking rate | 0.172 (0.053)* | 0.168 (0.052)* |
| Alcohol consumption | 0.113 (0.048)* | 0.108 (0.047)* |
| Urban (ref: rural) | –0.773 (0.512) | –0.746 (0.508) |
| *R²* | 0.581 | 0.489 |
| *F* | 41.5* | 36.8* |
| *Notes:* **P*<0.05. | | |
