# Supplementary material for: Mortality trends and influencing factors of mental disorders among urban and rural residents in China between 2009 and 2021: a longitudinal study with projections to 2030
Source: Front Public Health. 2026 Sep 3;14:1851033. doi: 10.3389/fpubh.2026.1851033 (PMC13581816; doi:10.3389/fpubh.2026.1851033)
Supplement: Supplementary file 1 [file Data_Sheet_1.ZIP › Supplementary Table/Supplementary table S5. Variable importance ranking based on standardized regression coefficients.docx]

**Supplementary table S5.** Variable importance ranking based on standardized regression coefficients

| Rank | Variable | Unstandardized *β* (95% *CI*) | Standardized *β* | *t* | *P* |
| --- | --- | --- | --- | --- | --- |
| 1 | Aging rate | 0.650 (0.493, 0.807) | 0.352 | 8.13 | <0.001 |
| 2 | Suicide-specific mortality | 0.350 (0.213, 0.487) | 0.217 | 5 | <0.001 |
| 3 | Mental disorder prevalence | 0.420 (0.234, 0.606) | 0.191 | 4.42 | <0.001 |
| 4 | Adult smoking rate | 0.180 (0.082, 0.278) | 0.156 | 3.60 | <0.001 |
| 5 | Per capita disposable income | -0.300 (-0.633, 0.033) | -0.134 | -1.76 | 0.078 |
| 6 | Psychiatrist workload | -0.001 (-0.001, -0.001) | -0.133 | -4.33 | 0.001 |
| 7 | Urban (ref: rural) | -0.800 (-1.780, 0.180) | -0.127 | -1.60 | 0.110 |
| 8 | Psychiatric bed density | -0.010 (-0.012, -0.008) | -0.125 | -10 | <0.001 |
| 9 | Alcohol consumption | 0.120 (0.032, 0.208) | 0.115 | 2.67 | 0.008 |
| 10 | Basic medical insurance coverage | -0.035 (-0.066, -0.004) | -0.099 | -2.19 | 0.028 |
| 11 | Psychotherapist density | -0.015 (-0.031, 0.001) | -0.091 | -1.88 | 0.060 |
| 12 | Psychiatrist density | -0.006 (-0.014, 0.002) | -0.07 | -1.50 | 0.134 |
| 13 | Urbanization rate | -0.020 (-0.047, 0.007) | -0.069 | -1.43 | 0.152 |
| 14 | Community health center density | -0.001 (-0.003, 0.001) | -0.045 | -1 | 0.317 |
